# Landscape Transformation and Disease Emergence

**DOI:** 10.3201/eid1105.AC1105

**Published:** 2005-05

**Authors:** Polyxeni Potter

**Affiliations:** * Centers for Disease Control and Prevention, Atlanta, Georgia, USA

**Keywords:** Art and science, emerging infectious diseases, Eugene von Guérard, Ferntree Gully, Dandenong Ranges, landscape, nature, dengue, mosquitoes

**Figure:**
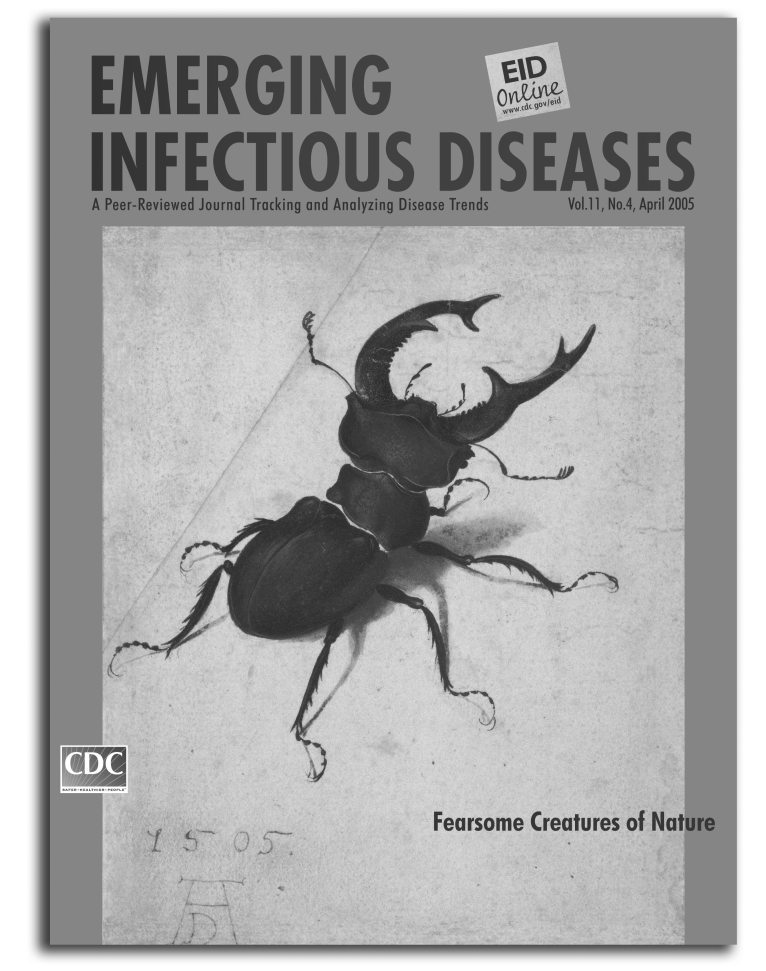
**Eugene von Guérard (1811–1901). Ferntree Gully in the Dandenong Ranges (1857).** Oil on canvas (92 cm × 138 cm). Gift of Dr. Joseph Brown AO OBE, 1975. National Gallery of Australia, Canberra, Australia

During his long ocean passage to Australia in 1852, Eugene von Guérard reported that conditions were far from ideal, and "meals were late and bad" (*1*). He had boarded a sailing ship at Gravesend, England, to seek his fortune in Victoria. At the gold fields of Ballarat, he described mining as "arduous at first" and causing "much backache and blistering of hands" (*1*). His gold mining efforts in Ballarat and environs were unsuccessful, and after a year, he abandoned the venture. Even so, he had struck gold. His illustrated diaries chronicled the history of the region, the harsh life of the gold digger, and the scarring of landscape from gold mining. He opened a studio in Melbourne, soon to become the most important Australian artist of his day.

The son of an artist and court painter, von Guérard was born in Vienna, Austria. He toured Italy with his father and lived in Rome for a while, where he became familiar with the work of famed French landscapists Claude Lorraine and Nicholas Poussin. Later, he studied landscape painting at the Dusseldorf Academy, where he was influenced by German romanticism—a movement that also dwelled on the visual aspects of nature. During his 30 years in Australia, he became a renowned landscape painter, as well as teacher and honorary curator at the National Gallery in Victoria. He died in London, where he had settled near the end of his life (*2*).

The 1850s gold rush that lured von Guérard to Victoria coincided with a revived interest in landscape painting, particularly in Australia and the United States (the Hudson River School). In the midst of 19th-century urbanization grew longing to connect with nature. Travelers sought areas of untouched wilderness, and artists labored to bring exotic freshness to the homebound. From his studio in Melbourne, von Guérard traveled to and explored many regions, among them timbered Illawarra and Tasmania, seeking the picturesque and generating drawings for his monumental landscape paintings (*3*).

At Illawarra, von Guérard was able to capture, in minute detail, the character of local flora: cabbage palms, ferns, fig trees, and multiple varieties of vines within the dark green tones of the dense Australian forest. Meticulous geographic, geologic, and ecosystem markers lend his artistic work historical importance as backdrop to subsequent transformation of the landscape by widespread mining and population growth.

In a letter to Melbourne newspaper The Argus in 1870, von Guérard explained that he painted "with the greatest desire to imitate nature" and sought to capture not only her details but also her "poetical feelings" (*4*). Descriptive and emotional, his elaborate artistic observations imbued the physical world with inner life quite apart from human society. Like many of his contemporaries (e.g., American painter Frederic Church), von Guérard was influenced by prominent German naturalist Alexander von Humboldt (1769–1859), who advocated a "mutual reinforcement of art and science." In this context, topographic detail was acceptable in paintings only if motivated and sustained by emotional connection and personal relevance. Landscape painting was a way to express love of nature. And nature was constantly changing, driven by forces that shaped it throughout the eons (*5*).

Ferntree Gully in the Dandenong Ranges on this month's cover was hailed a masterpiece in its day. A lush mountain panorama, this painting excels in its faithful depiction of both the forest and the trees. Balanced and lyrical, the work is a romantic rendering of pure, unadulterated nature. The scene is carefully structured: background fully outlined, center well-lit, and foreground intentionally shaded to frame and enhance view of the cloistered center. Each leaf is described in detail.

A broad path leads inward for a better look at the botanical life nearby as well as the treed horizon afar. A couple of lyrebirds walk the brush, their ancient silhouettes outlined against the grounds sheltering their fare of insects, myriapods, and snails. Curving fern tops and tree branches create a circular feeling as the eye moves from dark to light, from mountaintop to forest floor, from live greenery to fallen tree limbs and skeletal trunks, recounting a natural cycle of death and regeneration (*6*).

Von Guérard provided a respectful glimpse at unspoiled wilderness. His artistic eye scanned the exotic flora and through the bucolic stillness saw the real Arcadia, a goldmine of natural elements in constant change.

The semitropical rainforest idyll witnessed by von Guérard in Illawarra repeats itself in tropical and subtropical regions around the globe. Under a canopy of green, away from direct light, rain, and wind, moisture seeps down or hangs in mid-air, creating a fertile environment for propagation and growth. Along with fern spores, wildlife and microbial life are beneficiaries of the gullies' hothouse. Tiny creatures of the forest and blood-sucking insects, nature's fine detail, populate the underbrush—among them, mosquitoes, which feed on wild animals and thrive in this habitat.

As human development encroaches on the forest and urbanization transforms the native environment, mosquitoes become able to travel to all global destinations. These adaptable insects, some of them vectors of dengue viruses, have become anthropophilic, domesticated, and dangerous to humans. Environmental change affects nature's cycle, once more frustrating efforts to disrupt the persistent reemergence of tropical diseases like dengue (*7*).
